# Fibroblast Cell Responses to Vanadium and Niobium Titanium Alloys: A Biocompatibility Study

**DOI:** 10.1021/acsomega.3c04252

**Published:** 2023-09-08

**Authors:** Ayse Ak

**Affiliations:** Kocaeli Vocational School of Health Services, Department of Medical Services and Techniques, Medical Imaging Techniques Program, Kocaeli University, Kocaeli 41380, Turkey

## Abstract

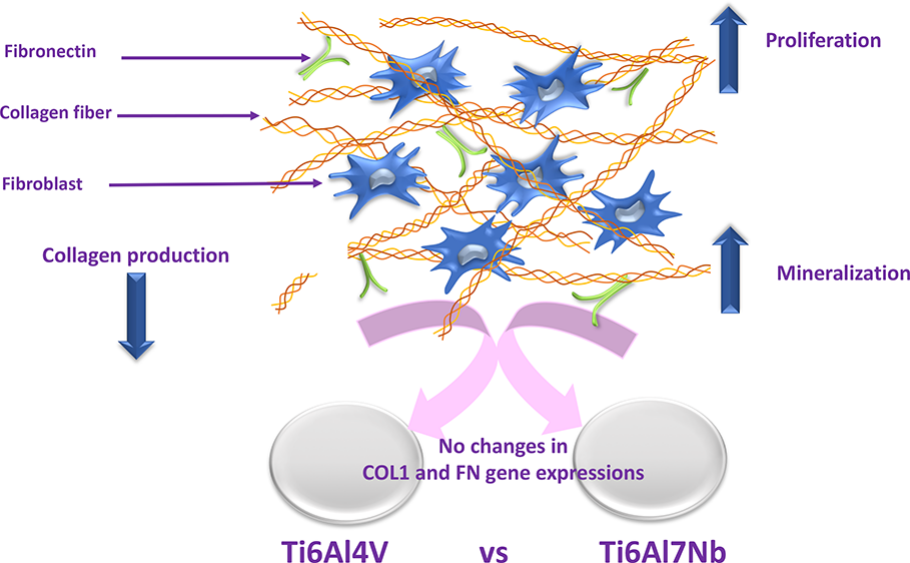

The interactions of a biomaterial with tissues must be determined for the material to be fully compatible with the body for a long time. The tissue and environment where the material is implanted are highly affected by its content. Titanium-6Aluminum-4Vanadium is widely used in orthopedics and dentistry. Recently, Titanium-6Aluminum-7Niobium alloys have been studied because of Titanium-6Aluminum-4Vanadium toxicity, which may be caused by vanadium. The aim of this study was to determine whether Titanium-6Aluminum-4Vanadium and Titanium-6Aluminum-7Niobium affect fibroblast cell proliferation, mineralization, and collagen production and whether they change the expression of type 1 collagen and fibronectin genes. It was determined that the niobium-containing alloy increased cell proliferation and calcium mineralization compared with the vanadium-containing alloy (*p* < 0.05). However, the alloys did not cause changes in the expression of collagen type 1 or fibronectin in cells. The collagen content of the cells on the niobium-containing alloy was lower than that on both the vanadium-containing alloy and tissue culture plate surface (*p* < 0.05). The niobium-containing alloy was found to be superior to the vanadium-containing alloy in terms of cell proliferation and calcium mineralization. Furthermore, neither vanadium-containing alloy nor niobium-containing alloy implant materials altered gene expression. Although both alloys are considered compatible with bone tissue, it should be considered whether they are also biocompatible with fibroblast cells.

## 1 Introduction

Titanium and its alloys are commonly used in orthopedics and dentistry and are typically expected to have a long-lasting life. However, implantation fails when it is not sufficiently biointegrated with the bone and/or surrounding tissues.^1^ After placing a biomaterial in the body, its interaction with the surrounding cells/tissues needs to be evaluated to determine whether the biomaterial is biocompatible and/or whether the healing process is successful.^2,3^

The biocompatibility of a material is determined by a number of functions such as proliferation, adhesion, and tissue formation of host body cells in contact with the material.^4,5^ Calcium, which is involved in many cellular functions, is a secondary messenger molecule that regulates cell proliferation, migration and cell death.^6,7^ Concurrently, the extracellular level of calcium is also important, as it is the main component of the extracellular matrix (ECM), mineralization, and osseointegration.^8^ Although many studies on orthopedic implants have focused on osseointegration before fibro integration^2,4,9^ soft-tissue formation around the implant is as critical as osseointegration. Collagen and fibronectin, which are large components of the ECM, also play a role in soft tissue formation and are produced by fibroblast cells.^10^ Collagen and fibronectin are involved in adhesion, proliferation, and differentiation processes.^9−13^

Vanadium and its derivatives are ultratrace elements that are structural analogues of phosphates and are thought to be essential for living organisms.^14^ Although vanadium-containing Titanium-6Aluminum-4Vanadium (Ti6Al4V) is the most commonly used alloy for medical implants, there are still many unresolved questions regarding the effects of its components on living tissue. Despite the excellent corrosion properties and mechanical strength of Ti6Al4V, the released metal ions may cause long-term problems,^15−18^ and also, titanium alloys with V content are more expensive than those without V.^17^ Recently, niobium-containing Ti alloys have been proposed as alternatives to Ti6Al4V.^18^ Titanium-6Aluminum-7Niobium (Ti6Al7Nb) has very similar physical properties to Ti6Al4V, showing improved corrosion resistance, and niobium (Nb) ions are considered less toxic than vanadium.^16−20^ However, there is limited information on the biological responses of the Ti6Al7Nb alloy.^21^

In the present study, fibroblasts were cultured on Ti6Al4V and Ti6Al7Nb surfaces *in vitro*, and the effects of Ti alloys on cell proliferation, calcium mineralization, and total collagen content were investigated. Furthermore, type 1 collagen (COL1) and fibronectin (FN) gene expressions were investigated to understand the functionalities of the cells.

## 2 Materials and Methods

### 2.1 Sample Preparation, Cell Culture, and Proliferation Assay

The commercially available Ti alloys used in this study are Ti6Al4V and Ti6Al7Nb in hot rolled and annealed discs with 10 mm diameter and 2 mm thickness. The chemical compositions (wt %) of materials provided by their manufacturers are given in Table 1. Specimens were ground with 220, 400, 800, 1200, and 2000 grade silicon carbide emery papers to obtain clean and homogeneous surfaces. The obtained average surface roughness (Ra) of materials were 0.48 ± 0.04 μm for Ti6Al4V and 0.51 ± 0.06 μm for Ti6Al7Nb. The discs were sterilized by autoclaving at 121 °C for 2 h.

**Table 1:** Chemical Compositions of Ti Alloys (% wt)

element	Ti6Al4V	Ti6Al7Nb
Al	6.14	5.81
V	3.92	
Nb		6.85
Fe	<0.3	<0.25
O	<0.2	<0.2
C	<0.08	<0.08
N	<0.05	<0.05
others	<0.04	<0.04
Ti	balance	balance

The mouse fibroblast cell line-L929 (ATCC CCL-1) was cultured in Dulbecco’s Modified Eagle’s Medium (Gibco,USA) supplemented with 10% fetal bovine serum (Sigma-Aldrich, St Louis, MO, USA), 2 mM glutamine (Sigma Chemical Co., St. Louis, MO, USA), and 1% penicillin/streptomycin solution (Gibco,USA) in a humidified atmosphere containing 5% CO_2_ at 37 °C. Fibroblasts were seeded on 24-well tissue culture plate (Sigma-Aldrich, St Louis, MO, USA) surfaces (TCPS) and Ti alloy discs at a density of 40,000 cells/well. The cytotoxicity assays were performed in six separate experiments (*n* = 6). Well plates containing the cells were incubated for 24, 48, and 72 h. TCPS was used in all experiments as control samples. Cell proliferation was measured using an Alamar blue assay. After each incubation period, the medium was replaced with fresh medium supplemented with 10% PBS containing resazurin sodium salt (R7017, Sigma, USA). After 3 h of incubation at 37 °C in a humidified atmosphere, the optical density of the medium was read at 570 nm using an automated microplate reader (Thermo Fisher Scientific, Waltham, MA, USA).

### 2.2 Mineralization Assay

Plates were washed with cold PBS and fixed for 1 h at 4 °C in cold 70% ethanol (sample size *n* = 9). After washing twice with sterile water, alizarin red solution (40 mM, pH 4.2) (Chembio, Germany) was added, and the cells were stained for 30 min.^22^ After staining, the wells were rinsed with PBS and a solution containing 15% acetic acid and 20% methanol was added to solubilize the precipitate. The alizarin red S should solubilize into the extraction buffer, resulting in a color change from red to yellow. The optical density of the solution was measured at 405 nm wavelength.^23^

### 2.3 Total Collagen Quantification Assay

The collagen content in cells (sample size *n* = 6) was measured using a Sirius Red/Fast Green collagen staining kit (Chondrex Inc., USA). The medium was removed, and the wells were washed with 1× PBS. One milliliter of Kahle fixative (60 mL of distilled water, 28 mL of 96% ethanol, 10 mL of 37% formaldehyde, and 2 mL of glacial acetic acid) was added, and the cells were incubated for 10 min at room temperature. A dye solution (0.2–0.3 mL) was added and incubated for 30 min at room temperature. The dye solution was aspirated, and the wells were rinsed with distilled water (0.5 mL of distilled water. A dye extraction buffer (1 mL) was added to each sample and mixed gently by pipetting until the color was eluted. The OD values were read at 540 and 605 nm using a spectrophotometer (Thermo Fisher Scientific, Waltham, MA, USA).

### 2.4 Type 1 Collagen and Fibronectin Gene Expressions

RNA was isolated from cells (*n* = 6) treated with alloys using a PureLink RNA Mini Kit (Thermo Fisher Scientific, USA). The purity and concentration of the isolated RNAs were determined using a spectrophotometer (Thermo MultiskanGo, Drop Plate, USA). Complementary DNA (cDNA) was obtained using the RT2 HT First Strand kit (Qiagen) using 100 ng/μL of RNA in cell groups.

cDNA synthesis was carried out using a PCR (Techne) device by incubating at 42 °C for 5 min, 42 °C for 15 min, and 95 °C for 5 min. Expression levels of type 1 collagen (COL1A1) and fibronectin genes were determined using a Magnetic Induction Cycler (Mic qPCR) device with an RT2 SYBR Green FAST Mastermix kit.

The qRT-PCR conditions were as follows: 1 cycle at 95 °C for 10 min, 40 cycles at 95 °C for 15 s, 55 °C for 35 s, and 72 °C for 30 s. The qRT-PCR reaction was prepared using 12.5 μL of Mastermix, 1.25 μL of forward primer (10 μM), 1.25 μL of reverse primer (10 μM), 2 μL of cDNA, and 8 μL of nuclease-free water. F-R- primer sequences were used for fibronectin, while F-R- primer sequences were used to determine collagen gene expression.

The GAPDH gene was selected as the housekeeping gene, and F-R- sequences were used. Target genes were normalized to GAPDH gene and gene expression levels, and Cq values were calculated according to the formula 2^–ΔΔCt^ = 2^(–(treated Ct target gene – treated Ct housekeeping gene) – (untreated Ct target gene – untreated Ct housekeeping gene)).^24^

The control group that was not incubated with the alloy was used as the calibrator, and the gene expression level was calculated according to this group. No template control (NTC) was read that did not contain the cDNA template in each read performed by qRT-PCR.

### 2.5 Statistical Analysis

One-way analysis of variance (ANOVA) was applied, and a Tukey-b test was used for multiple comparisons. Statistical calculations were conducted using the IBM SPSS 26 program (SPSS Inc., Chicago, IL, USA). Values were normalized to the tissue culture polystyrene surface control and represented as a percentage of the mean ± standard deviation (SD). The mean difference was significant at the level of 0.05.

## 3 Results

**Figure 1:**
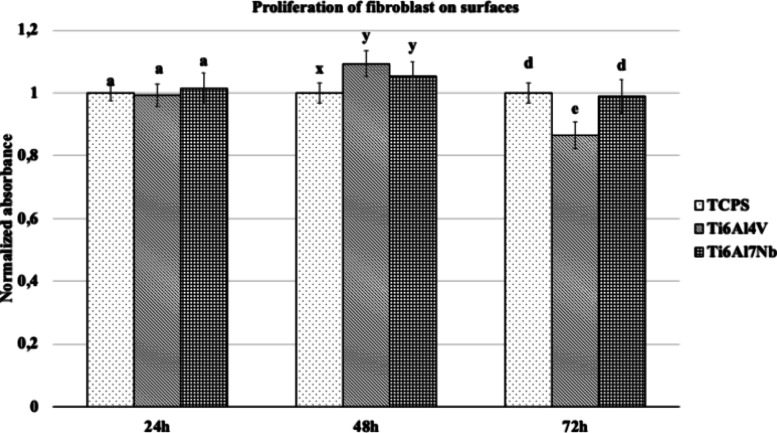
Proliferation of fibroblasts on Ti alloys and TCPS for 24, 48, and 72 h (*n* = 6). Bars indicate the standard deviation. Groups at each hour with different letters are statistically different (*p* < 0.05).

An Alamar blue assay was used to measure the effect of surfaces on fibroblasts, and the proliferation on surfaces is presented in Figure 1. Although the alloys used did not support cell proliferation at 24 h, they promoted proliferation at 48 h compared to the control. At 72 h, the niobium-containing alloy surface TCPS had a significantly greater proliferative effect than the vanadium-containing Ti alloy (*p* < 0.05).

**Figure 2:**
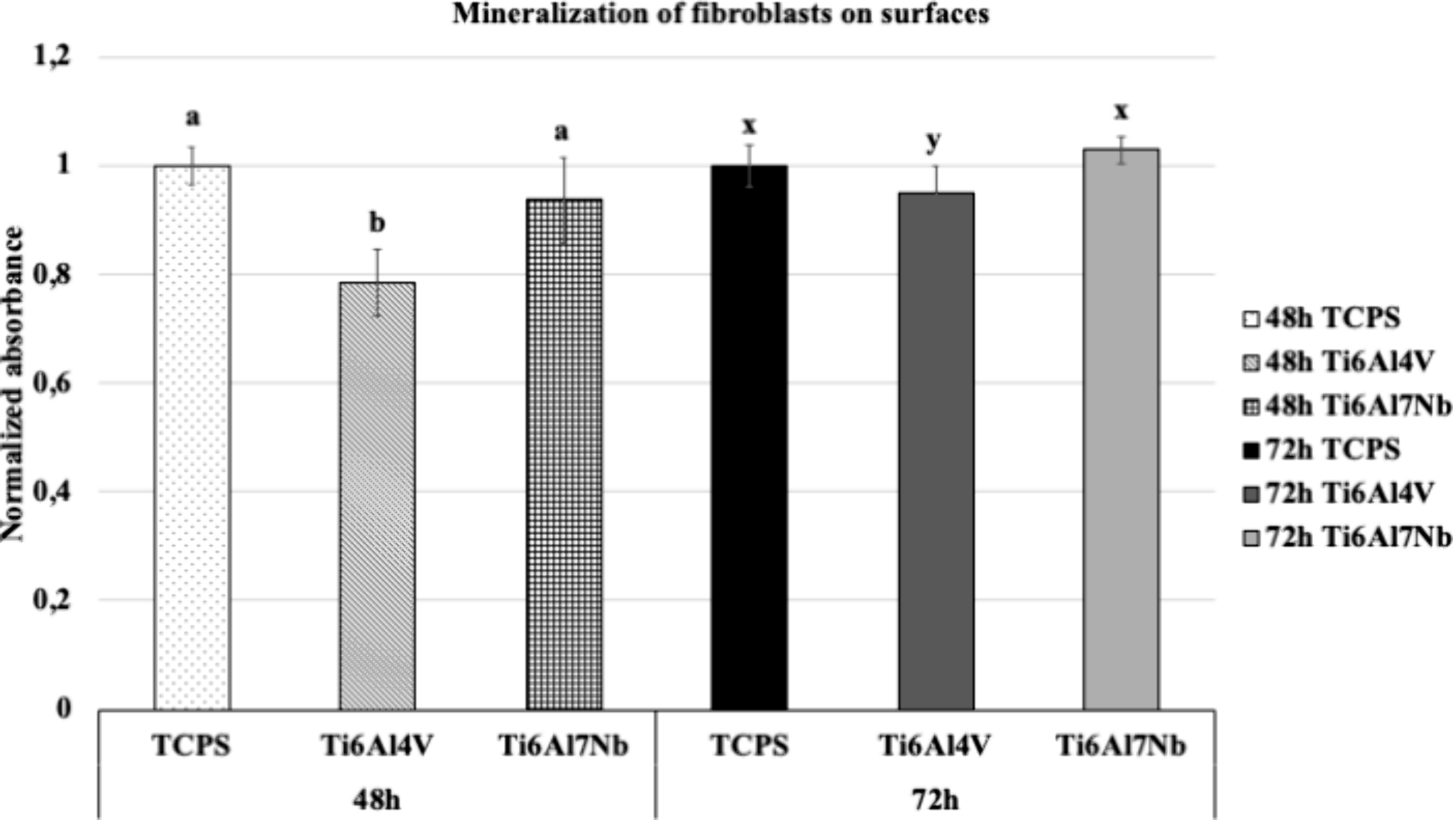
Calcium mineralization of fibroblast cells on Ti alloys and TCPS after 48 and 72 h (*n* = 9). Bars indicate the standard deviation. Groups in each hour with the different letters are statistically different (*p* < 0.05).

Alizarin red quantification indicated that the calcium mineralization of the cells on Ti6Al4V was statistically lower at the 48th and 72nd hours compared to Ti6Al7Nb and TCPS (*p* < 0.05). In contrast, the mineralization of cells on TCPS and niobium-containing surfaces was similar (Figure 2).

**Figure 3:**
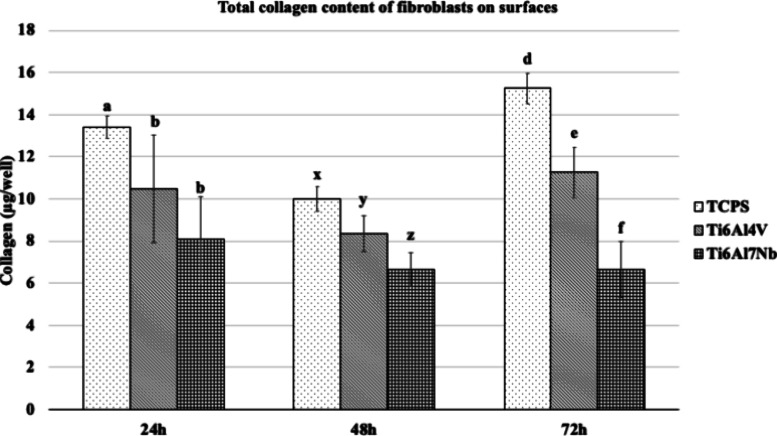
Total collagen content of fibroblasts on Ti alloys and TCPS (*n* = 6). Bars indicate the standard deviation. Groups in each hour with the different letters are statistically different (*p* < 0.05).

When the total collagen secreted by fibroblast cells was examined, it was determined that the niobium-containing Ti alloy had less collagen content than the TCPS and vanadium-containing Ti alloy at all time points (Figure 3).

**Figure 4:**
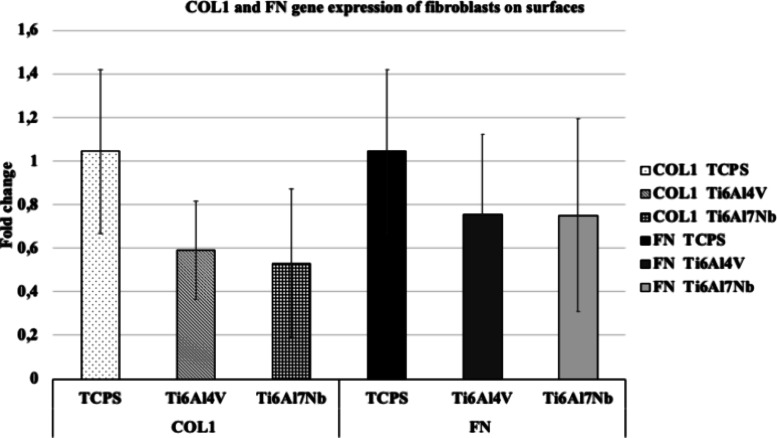
COL1 and FN gene expression in fibroblasts on Ti alloys and TCPS for 72 h (*n* = 6).

As shown in Figure 4, there was no significant difference in the expression of COL1 and FN at 72 h between the groups.

## 4 Discussion

Titanium alloys, which are frequently used in biomedical applications, have high biocompatibility, low density, corrosion resistance, and high-strength properties.^17,25,26^ In addition, metallic implants release potentially hazardous substances, thus diminishing the biocompatibility of the materials. The release of toxic ions into the body triggers an inflammatory reaction in the surrounding tissue, which can lead to implant discoloration and loosening. Consequently, preventing the release of toxic ions is crucial for ensuring the well-being of patients.^27−29^ Although Ti6Al4V is the most commonly used implant, it may cause long-term toxicity and implant rejection because it contains vanadium.^14,30^ According to ISO 10993-5-2009, vanadium-containing Ti alloys are not considered cytotoxic as they do not cause more than 30% cell death.^31^ Commercially pure titanium, which was unsuccessful clinically, led to the development of Ti6Al4V. However, this alloy containing vanadium can also result in toxicity during prolonged usage. Therefore, the Ti6Al7Nb alloy was developed to enhance mechanical properties while avoiding such toxicity, but there is not enough information about their biological activity, and niobium-containing Ti alloys do not have a wide usage area.^32,33^

It is insufficient to evaluate the compatibility of a material to be implanted in hard tissue based only on bone tissue. Compatibility with fibroblast cells, which constitute a large part of the surrounding tissues, should also be considered. It was also reported that exposing cells to a higher level of extracellular calcium concentration or calcium-enriched materials would stimulate the recruitment, proliferation, differentiation, and bone-forming capacity of these cells.^34^ Based on our study, it is observed that the niobium-containing Ti alloy is more biocompatible compared to the vanadium-containing alloy as it stimulates higher fibroblast proliferation and calcium mineralization. Similar to the results of the present study, it was found in different cell lines that surfaces containing Nb caused more preosteoblast cell proliferation^28^ and stimulate the viability and calcium mineralization of mesenchymal stem cells.^31^ It has also been determined that niobium-containing different alloys such as Ti-Nb-Zr-Ta and Ti-Nb-Zr-Al lead to higher fibroblast cell viability compared to vanadium-containing Ti alloys.^41^

However, contrary to these studies and our results, it has been reported that these two Ti alloy surfaces have no effect on proliferation of human osteosarcoma cells,^35−37^ mesenchymal stem cells,^19^ Saos-2 osteoblasts, and EA.hy-926 endothelial cells,^38^ and even alloys containing Nb cause cytotoxicity in Saos cells.^39^ Of the two alloys containing nanotube films, it has been reported that alloys containing vanadium have better osteoblastic activities.^40^ Another study with titanium alloys containing molybdenum and chromium reported that these alloys caused slight cytotoxicity in fibroblast cells due to the release of elements into the environment. Particularly, prolonged exposure to molybdenum released from Ti-10Mo-10Cr resulted in reduced cell viability.^41^ The exposure duration is also important along with the elements contained in the titanium alloy.

There are 19 types of collagen, which is the main component of all connective tissues. Physical and chemical damage to tissue disrupts the organization of collagen.^42−44^ Collagen production has been demonstrated in many cell types cultured on Ti alloys,^45−47^ and total collagen, determined by Sirius red dye, which can bind to all collagen types,^42,43^ was synthesized on both alloys. However, similar to the study by Lochner,^48^ collagen synthesis was significantly lower on both alloys than on the TCP surface. Type 1 collagen (COL1), on the other hand, is a type of collagen synthesized by fibroblasts and osteoblasts, forming a large part of the total collagen.^49^ Niobium-containing alloys were found to be effective in stimulating COL1, osteopontin, and FN gene expression,^50,51^ whereas vanadium-containing alloys did not affect COL 1 and osteopontin expression.^52^ However, the Ti alloys used in this study did not change the gene expression of the fibroblast cells. These conflicting results may be due to different cell types and incubation times^28,38,53^ and different releases of vanadium and niobium into biological fluids (medium, calf serum, and PBS).^29,54^

## 5 Conclusions

Most studies that investigated the biocompatibility of Ti alloys have focused on their cytotoxicity, adhesion, and proliferation effects. In this study, the amounts of calcium mineralization and total collagen were determined in addition to proliferation. Furthermore, the expression of the COL1 and FN genes was evaluated. While fibroblast cell proliferation and mineralization were stimulated by a niobium-containing Ti alloy, it was observed that the collagen amount was lower than the vanadium-containing Ti alloy. However, both alloys did not affect the gene expression levels. Although vanadium is a biological trace element, vanadium-containing Ti alloys may not have superior biological and functional properties. Additionally, niobium-containing Ti alloys can be considered as alternatives to Ti6Al4V for biomedical applications. Also, Ti6Al7Nb implants may be more effective than Ti6Al4V implants in terms of cell proliferation and mineralization. It is accepted that both implant materials are conducive to cell growth and are compatible with bone tissue. However, the effectiveness of fibroblasts, which constitute the surrounding tissues, in the biocompatibility process should also be known. It is important to consider the type of implant and the purpose of its application when making such comparisons.

## References

[ref1] Ehlert M.; Radtke A.; Jędrzejewski T.; Roszek K.; Bartmański M.; Piszczek P. In vitro studies on nanoporous, nanotubular and nanosponge-like titania coatings, with the use of adipose-derived stem cells. Materials 2020, 13, 1574 10.3390/ma13071574. 32235354 PMC7177883

[ref2] Advincula M.-C.; Rahemtulla F.-G.; Advincula R.-C.; Ada E.-T.; Lemons J.-E.; Bellis S.-L. Osteoblast adhesion and matrix mineralization on sol–gel-derived titanium oxide. Biomaterials 2006, 27 (10), 2201–2212. 10.1016/j.biomaterials.2005.11.014. 16313951

[ref3] Gil J.; Manero J.-M.; Ruperez E.; Velasco-Ortega E.; Jiménez-Guerra A.; Ortiz-García I.; Monsalve-Guil L. Mineralization of titanium surfaces: Biomimetic implants. Materials 2021, 14, 2879 10.3390/ma14112879. 34072082 PMC8198012

[ref4] Cortizo M.-C.; de Mele M.F.-L.; Cortizo A.-M. Metallic dental material biocompatibility in osteoblast like cells. Biol. Trace Elem. Res. 2004, 100, 151 10.1385/BTER:100:2:151. 15326364

[ref5] Hanawa T. Titanium–tissue interface reaction and its control with surface treatment. Front. Bioeng. Biotechnol. 2019, 7, 170 10.3389/fbioe.2019.00170. 31380361 PMC6650641

[ref6] Berridge M.-J The inositol trisphosphate/calcium signaling pathway in health and disease. Physiol. Rev. 2016, 96 (4), 1261–1296. 10.1152/physrev.00006.2016. 27512009

[ref7] Cui C.; Merritt R.; Fu L.; Pan Z. Target in calcium signaling in cancer therapy. Acta Pharm. Sin. B 2017, 7 (1), 3–17. 10.1016/j.apsb.2016.11.001. 28119804 PMC5237760

[ref8] Maeda M.; Hirose M.; Ohgushi H.; Kirita T. In vitro mineralization by mesenchymal stem cells cultured on titanium scaffolds. J. Biochem. 2007, 141 (5), 729–736. 10.1093/jb/mvm077. 17383975

[ref9] Bierbaum S.; Hempel U.; Geißler U.; Hanke T.; Scharnweber D.; Wenzel K.-W.; Worch H. Modification of Ti6AL4V surfaces using collagen I, III, and fibronectin. II. Influence on osteoblast responses. J. Biomed. Mater. Res., Part A 2003, 67A (2), 431–438. 10.1002/jbm.a.10084. 14566783

[ref10] Guillem-Marti J.; Delgado L.; Godoy-Gallardo M.; Pegueroles M.; Herrero M.; Gil F.-J. Fibroblast adhesion and activation onto micro-machined titanium surfaces. Clin. Oral Implants Res. 2013, 24 (7), 770–80. 10.1111/j.1600-0501.2012.02451.x. 22458450

[ref11] Ma H.-P.; Chang H.-L.; Bamodu O.-A; Yadav V.-K.; Huang T.-Y.; Wu A.T.-H; Yeh C.-T.; Tsai S.-H.; Lee W.-H. Collagen 1A1 (COL1A1) is a reliable biomarker and putative therapeutic target for hepatocellular carcinogenesis and metastasis. Cancers 2019, 11 (6), 786 10.3390/cancers11060786. 31181620 PMC6627889

[ref12] Smith P.-C; Martínez C.; Martínez J.; McCulloch C.-A. Role of fibroblast populations in periodontal wound healing and tissue remodeling. Front. Physiol. 2019, 10 10.3389/fphys.2019.00270. 31068825 PMC6491628

[ref13] Paganelli A.; Benassi L.; Rossi E.; Magnoni C. Extracellular matrix deposition by adipose-derived stem cells and fibroblasts: a comparative study. Arch. Dermatol. Res. 2020, 312, 295–299. 10.1007/s00403-019-01997-8. 31616972

[ref14] Treviño S.; Díaz A.; Sánchez-Lara E.; Sanchez-Gaytan B. L.; Perez-Aguilar J. M.; González-Vergara E. Vanadium in biological action: Chemical, pharmacological aspects, and metabolic mplications in diabetes mellitus. Biol. Trace Elem. Res. 2019, 188 (1), 68–98. 10.1007/s12011-018-1540-6. 30350272 PMC6373340

[ref15] Metikos̆-Huković M.; Kwokal A.; Piljac J. The influence of niobium and vanadium on passivity of titanium-based implants in physiological solution. Biomaterials 2003, 3765 10.1016/s0142-9612(03)00252-7. 12818549

[ref16] Shapira L.; Klinger A.; Tadir A.; Wilensky A.; Halabi A. Effect of a niobium-containing titanium alloy on osteoblast behavior in culture. Clin. Oral Implants Res. 2009, 20 (6), 578–582. 10.1111/j.1600-0501.2009.01707.x. 19530314

[ref17] Senopati G.; Rahman Rashid R. A.; Kartika I.; Palanisamy S. Recent Development of Low-Cost β-Ti Alloys for Biomedical Applications: A Review. Metals 2023, 13, 194 10.3390/met13020194.

[ref18] Falanga A.; Laheurte P.; Vahabi H.; Tran N.; Khamseh S.; Saeidi H.; Khodadadi M.; Zarrintaj P.; Saeb M. R.; Mozafari M. Niobium-treated titanium implants with improved cellular and molecular activities at the tissue-implant interface. Materials 2019, 22 (23), 3861 10.3390/ma12233861. PMC6926753 31766663

[ref19] Ureña J.; Gordo E.; Ruiz-Navas E.; Vilaboa N.; Saldaña L.; Jiménez-Morales A. Electrochemical comparative study on corrosion behavior of conventional and powder metallurgy titanium alloys in physiological conditions. Metal Powder Rep. 2017, 72 (2), 118–123. 10.1016/j.mprp.2016.04.003.

[ref20] Nune K.; Kumar A.; Misra R.; Li S.; Hao Y.; Yang R. Osteoblast functions in functionally graded Ti-6Al-4 V mesh structures. J. Biomater. Appl. 2016, 30 (8), 1182–1204. 10.1177/0885328215617868. 26637443

[ref21] Huang H.-H.; Wu C.-P.; Sun Y.-S.; Lee T.-H. Improvements in the corrosion resistance and biocompatibility of biomedical Ti–6Al–7Nb alloy using an electrochemical anodization treatment. Thin Solid Films 2013, 528, 157–162. 10.1016/j.tsf.2012.08.063.

[ref22] 22.Marshall C. D.; Brett E. A.; Moore A. L.; Wan D. C.; Longaker M. T. In Vitro and In Vivo Osteogenic Differentiation of Human Adipose-Derived Stromal Cells. Methods Mol. Biol. 2019, 1891, 9–18. 10.1007/978-1-4939-8904-1_2. 30414122 PMC7159064

[ref23] Gregory C. A.; McNeill E. P.; Pan S. Preparation of osteogenic matrices from cultured cells. Methods Cell Biol. 2020, 15–43. 10.1016/bs.mcb.2019.10.009. PMC7444597 32222217

[ref24] Schmittgen T.-D; Livak K.-J. Analyzing real-time PCR data by the comparative C T method. Nat. Protoc. 2008, 3 (6), 1101 10.1038/nprot.2008.73. 18546601

[ref25] Abdel-Hady Gepreel M.; Niinomi M. Biocompatibility of Ti-alloys for long-term implantation. J. Mech. Behav. Biomed. Mater. 2013, 20, 407–415. 10.1016/j.jmbbm.2012.11.014. 23507261

[ref26] Nicholson J. W. Titanium alloys for dental implants: A review. Prosthesis 2020, 2, 100–116. 10.3390/prosthesis2020011.

[ref27] Koike M.; Lockwood P.-E.; Wataha J.-C.; Okabe T. Initial cytotoxicity of novel titanium alloys. J. Biomed Mater. Res. Part B: Appl. Biomater 2007, 83B, 327–331. 10.1002/jbm.b.30799. 17385227

[ref28] Challa V.S.-A; Mali S.; Misra R.D.-K. Reduced toxicity and superior cellular response of preosteoblasts to Ti-6Al-7Nb alloy and comparison with Ti-6Al-4V. J. Biomed. Mater. Res., Part A 2013, 101A (7), 2083–2089. 10.1002/jbm.a.34492. 23349101

[ref29] Oktay A.; Yilmazer H.; Przekora A.; Yilmazer Y.; Wojcik M.; Dikici B.; Ustundag C.-B. Corrosion response and biocompatibility of graphene oxide (GO)serotonin (Ser) coatings on Ti6Al7Nb and Ti29Nb13Ta4.6Zr (TNTZ) alloys fabricated by electrophoretic deposition (EPD). Mater. Today Commun. 2023, 105236 10.1016/j.mtcomm.2022.105236.

[ref30] Ścibior A.; Pietrzyk L.; Plewa Z.; Skiba A. Vanadium: Risks and possible benefits in the light of a comprehensive overview of its pharmacotoxicological mechanisms and multi-applications with a summary of further research trends. J. Trace Elements Med. Biol. 2020, 126508 10.1016/j.jtemb.2020.126508. PMC7152879 32305626

[ref31] ISO 10993–5:2009. Biological Evaluation of Medical Devices—Part 5: Tests for *In* Vitro Cytotoxicity; International Organization for Standardization: Geneva, Switzerland, 2009.

[ref32] Osathanon T.; Bespinyowong K.; Arksornnukit M.; Takahashi H.; Pavasant P. Human osteoblast-like cell spreading and proliferation on Ti-6Al-7Nb surfaces of varying roughness. J. Oral Sci. 2011, 53 (1), 23–30. 10.2334/josnusd.53.23. 21467811

[ref33] Prosolov K.-A.; Mitrichenko D.-V.; Prosolov A.-B.; Nikolaeva O.-O.; Lastovka V.-V.; Belyavskaya O.-A.; Chebodaeva V.-A.; Glukhov I.-A.; Khlusov I.-A.; Litvinova L.-S.; Shupletsova V.-V.; Khaziakmatova O.-G.; Malashchenko V.-V.; Yurova K.-A.; Shunkin E.-O.; Fedorov M.-A.; Komkov A.-R.; Pavlenko V.-V.; Anisenya I.-I.; Sharkeev Y.-P; Vladescu A.; Khlusov I.-A. Zn-Doped CaP-Based Coatings on Ti–6Al–4V and Ti–6Al–7Nb alloys prepared by magnetron sputtering: controllable biodegradation, bacteriostatic, and osteogenic activities. Coatings 2021, 809 10.3390/coatings11070809.

[ref34] Minghan C.; Na L.; Sharma N.; Li W.; Chen C.; Dong B.; Cheng L.; Wang L.; Thieringer F.-M. Positive regulation of osteogenesis on titanium surface by modification of nanosized Ca2+-exchanged EMT zeolites. Mater. Today Commun. 2022, 104874 10.1016/j.mtcomm.2022.104874.

[ref35] Bărbînţă A. C.; Earar K.; Crimu C. I.; Drăgan L. A.; Munteanu C. In Vitro Evaluation of the Cytotoxicity of Some New Titanium Alloys. Key Eng. Mater. 2013, 587, 303–308. 10.4028/www.scientific.net/KEM.587.303.

[ref36] Bărbînţă A.-C.; Earar K.; Crimu C.-I.; Drăgan L.-A.; Munteanu C. *In vitro* evaluation of the cytotoxicity of some new titanium alloys. Key Eng. Mater. 2013, 587, 303–308. 10.4028/www.scientific.net/KEM.587.303.

[ref37] Li H.; Zheng Y.; Lin J. Comparative Evaluation on the *In vitro* biological performance of Ti45Al8.5Nb intermetallic with Ti6Al4V and Ti6Al7Nb alloys. Adv. Eng. Mater. 2011, 13 (5), B187–B193. 10.1002/adem.201080110.

[ref38] Walkowiak-Przybyło M.; Komorowski P.; Walkowiak B. Differences in the expression of cell cycle genes in osteoblasts and endothelial cells cultured on the surfaces of Ti6Al4V and Ti6Al7Nb alloys. J. Biomed. Mater. Res., Part A 2017, 105 (6), 1607–1617. 10.1002/jbm.a.35972. 28002653

[ref39] El-Hadad S.; Safwat E.-M.; Sharaf N.-F. *In-vitro* and *in-vivo*, cytotoxicity evaluation of cast functionally graded biomaterials for dental implantology. Mater. Sci. Eng.: C 2018, 93, 987 10.1016/j.msec.2018.09.003. 30274137

[ref40] Stan M.-S.; Memet I.; Fratila C.; Krasicka-Cydzik E.; Roman I.; Dinischiotu A. Effects of titanium-based nanotube films on osteoblast behavior in vitro. J. Biomed. Mater. Res., Part A 2015, 103 (1), 48–56. 10.1002/jbm.a.35148. 24639011

[ref41] Mat-Baharin N. H.; Razali M.; Mohd-Said S.; Syarif J.; Muchtar A. Influence of alloying elements on cellular response and in-vitro corrosion behavior of titanium-molybdenum-chromium alloys for implant materials. J. Prosthodontic Res. 2020, 2020, 490 10.1016/j.jpor.2020.01.004. 32063537

[ref42] 42.Tullberg-Reinert H.; Jundt G. In situ measurement of collagen synthesis by human bone cells with a sirius red-based colorimetric microassay: effects of transforming growth factor beta2 and ascorbic acid 2-phosphate. Histochem Cell Biol. 1999, 112 (4), 271–276. 10.1007/s004180050447. 10550611

[ref43] Bhutda S.; Surve M.-V.; Anil A.; Kamath K.; Singh N.; Modi D.; Banerjee A. Histochemical staining of collagen and identification of its subtypes by picrosirius red dye in mouse reproductive tissues. Bio Protoc 2017, e2592 10.21769/BioProtoc.2592. PMC8438400 34595270

[ref44] Khalilimofrad Z.; Baharifar H.; Asefnejad A.; Khoshnevisan K. Collagen type I cross-linked to gelatin/chitosan electrospun mats: Application for skin tissue engineering. Mater. Today Commun. 2023, 105889 10.1016/j.mtcomm.2023.105889.

[ref45] Brie I.-C.; Soritau O.; Dirzu N.; Berce C.; Vulpoi A.; Popa C.; Todea M.; Simon S.; Perde-Schrepler M.; Virag P.; Barbos O.; Chereches G.; Berce P.; Cernea V. Comparative in vitro study regarding the biocompatibility of titanium-base composites infiltrated with hydroxyapatite or silicatitanate. J. Biol. Eng. 2014, 1 10.1186/1754-1611-8-14. 24987458 PMC4077223

[ref46] Lucaciu O.; Soriţău O.; Gheban D.; Ciuca D.-R.; Virtic O.; Vulpoi A.; Dirzu N.; Campian R.; Baciut G.; Popa C.; Simon S.; Berce P.; Baciut M.; Crisan B. Dental follicle stem cells in bone regeneration on titanium implants. BMC Biotechnol. 2015, 1 10.1186/s12896-015-0229-6. 26718927 PMC4697321

[ref47] Wang Y.; Lou J.; Zeng L.; Xiang J.; Zhang S.; Wang J.; Xiong F.; Li C.; Zhao Y.; Zhang R. Osteogenic potential of a novel microarc oxidized coating formed on Ti6Al4V alloy. Appl. Surf. Sci. 2017, 412, 29–36. 10.1016/j.apsusc.2017.03.191.

[ref48] Lochner K. The potential role of human osteoblasts for periprosthetic osteolysis following exposure to wear particles. Int. J. Mol. Med. 2011, 1055 10.3892/ijmm.2011.778. 21850366

[ref49] Calvi E. N. d. C.; Nahas F. X.; Barbosa M. V.; Calil J. A.; Ihara S. S. M.; Silva M. d. S.; Franco M. F. d.; Ferreira L. M. An experimental model for the study of collagen fibers in skeletal muscle. Acta Cir. Bras. 2012, 27 (10), 681–686. 10.1590/S0102-86502012001000003. 23033128

[ref50] Osathanon T.; Bespinyowong K.; Arksornnukit M.; Takahashi H.; Pavasant P. Ti-6Al-7Nb promotes cell spreading and fibronectin and osteopontin synthesis in osteoblast-like cells. J. Mater. Sci.: Mater. Med. 2006, 17 (7), 619–625. 10.1007/s10856-006-9224-8. 16770546

[ref51] Sun Y.-S.; Liu J.-F.; Wu C.-P.; Huang H.-H. Nanoporous surface topography enhances bone cell differentiation on Ti–6Al–7Nb alloy in bone implant applications. J. Alloys Compd. 2015, 10.1016/j.jallcom.2015.01.

[ref52] Jäger M.; Urselmann F.; Witte F.; Zanger K.; Li X.; Ayers D. C.; Krauspe R. Osteoblast differentiation onto different biometals with an endoprosthetic surface topography *in vitro*. J. Biomed. Mater. Res., Part A 2008, 86A (1), 61–75. 10.1002/jbm.a.31552. 17941017

[ref53] Costa B. C.; Tokuhara C. K.; Rocha L. A.; Oliveira R. C.; Lisboa-Filho P. N.; Costa Pessoa J. Vanadium ionic species from degradation of Ti-6Al-4V metallic implants: *In vitro* cytotoxicity and speciation evaluation. Mater. Sci. Eng.: C 2019, 96, 730–739. 10.1016/j.msec.2018.11.090. 30606586

[ref54] Okazaki Y.; Gotoh E. Comparison of metal release from various metallic biomaterials *in vitro*. Biomaterials 2005, 26, 11–21. 10.1016/j.biomaterials.2004.02.005. 15193877

